# Synergistic negative effects of thermal stress and altered food resources on echinoid larvae

**DOI:** 10.1038/s41598-018-30572-w

**Published:** 2018-08-15

**Authors:** Colette J. Feehan, Zoe Ludwig, Suzannah Yu, Diane K. Adams

**Affiliations:** 1 Department of Biology, Montclair State University, Montclair, 07043 USA; 2 Friday Harbor Laboratories, University of Washington, Friday Harbor, 98250 USA; 3 Department of Marine and Coastal Sciences, Rutgers University, New Brunswick, 08901 USA

## Abstract

Multiple changes to the marine environment under climate change can have additive or interactive (antagonistic or synergistic) effects on marine organisms. Prompted by observations of anomalously warm sea temperatures and low chlorophyll concentrations during the 2013–2016 warm “Blob” event in the Northeast Pacific Ocean, we examined the combined effects of thermal stress and a shift in food resources on the development of a larval echinoid (*Strongylocentrotus droebachiensis*) in the laboratory. A high concentration of phytoplankton yielded faster echinus rudiment development at warm versus historical temperature, indicating a mitigating effect of abundant food on thermal stress; however, low phytoplankton concentration or a shift in diet to suspended kelp detritus, yielded slow development and high mortality at warm temperature. The results indicate a synergistic negative effect of thermal stress and altered food resources on larvae of a keystone marine species.

## Introduction

Under climate change, extreme events such as heatwaves are predicted to increase in frequency and intensity^1^. One such event occurred in the Northeast Pacific from late 2013 through 2016, resulting in anomalously warm sea surface temperatures and low sea level pressure off the west coast of North America. A resultant, persistent mass of anomalously warm surface water was termed the “Blob” by ocean scientists^2^. The phenomenon led to changes in ocean biology and ecology including diet-shifts and mass-starvation of seabirds, and observations of tropical and subtropical species, such as ocean sunfish (*Mola mola*), north of their typical geographic range^3^. Additionally, in the winter and spring of 2014, researchers measured the lowest ocean chlorophyll concentrations observed since 1997, indicating diminished phytoplankton productivity^4^.

Combined stressors of warm sea temperature and low phytoplankton concentration may impact the planktonic larval stage of benthic marine invertebrates, such as sea urchins, that rely on phytoplankton as a source of food^5^. On both sides of the North Atlantic and in the Northeast Pacific, recruitment pulses of green sea urchins *Strongylocentrotus droebachiensis* into kelp beds have led to destructive grazing of kelps, and ecosystem phase shifts from kelp beds to sea urchin barrens^6–10^. This ecological phase shift is considered a “collapse” of the kelp ecosystem^11,12^. The impact of heatwaves on recruitment success of *S. droebachiensis* remains largely unresolved, but has important implications for our ability to predict destructive grazing events and subsequent changes to the structure and functioning of kelp ecosystems. Discordance among studies in terms of an upper thermal threshold for development and survival of larval *S. droebachiensis* indicates that populations in discrete regions with barriers to dispersal of planktonic larvae may differ in their tolerance to ocean warming^13–15^. In a study of larvae generated from *S. droebachiensis* collected at Sandy Cove, Nova Scotia, Canada, Hart and Scheibling^13^ found that growth rates of larvae increased with temperature from 3 to 9 °C, and were greatest at 14 °C. These results were consistent with observations of a strong sea urchin recruitment pulse in the late 1960s that led to destructive grazing of kelps following anomalously warm spring sea temperatures^13^. By contrast, Stephens^14^ studied *S. droebachiensis* larvae generated from individuals collected at Cape Cod Bay, Massachusetts, USA, and found that larval development declined above a thermal threshold of ~10 °C. Similar to Stephens, Fagerli *et al*.^15^ identified a correlation between recruitment failure of *S. droebachiensis* populations along the mid-coast of Norway and unusually warm sea temperatures (>10 °C). They argued that warming temperatures were limiting larval supply due to temperature-mediated larval mortality^15^.

The effects of multiple stressors on marine organisms can be examined in laboratory microcosm experiments through simultaneous manipulation of multiple factors, and may yield additive, antagonistic, or synergistic outcomes^16^. Under climate change, combinations of abiotic stressors resulting from multiple changes to the marine environment (e.g. temperature, salinity, and pH) often result in synergistic negative effects on marine invertebrate larvae^17^. Here, we test the effects of multiple stressors on larvae of *S. droebachiensis* by simulating conditions during the “Blob” marine heatwave. We reared larvae in the laboratory under conditions of historical (9 °C) and warm (17 °C) temperatures observed before and during the heatwave, respectively, in combination with high and low rations of phytoplankton food. Additionally, we included a third factor of food type, providing larvae with either phytoplankton or a suspended kelp-derived detritus diet (high and low ration), to examine whether locally abundant, kelp-derived particulate organic matter^18,19^ provides a suitable diet for larvae at warm temperature in the absence of abundant phytoplankton. We hypothesized that thermal stress and limited availability of food would combine synergistically to reduce larval development and survival. Based on previous observations that kelp detritus rivals a phytoplankton diet in terms of food quality for larval *S. droebachiensis* at 9 °C^20^, we predicted that abundant kelp detritus would be a suitable substitute for phytoplankton food under thermally stressed conditions.

**Figure 1:**
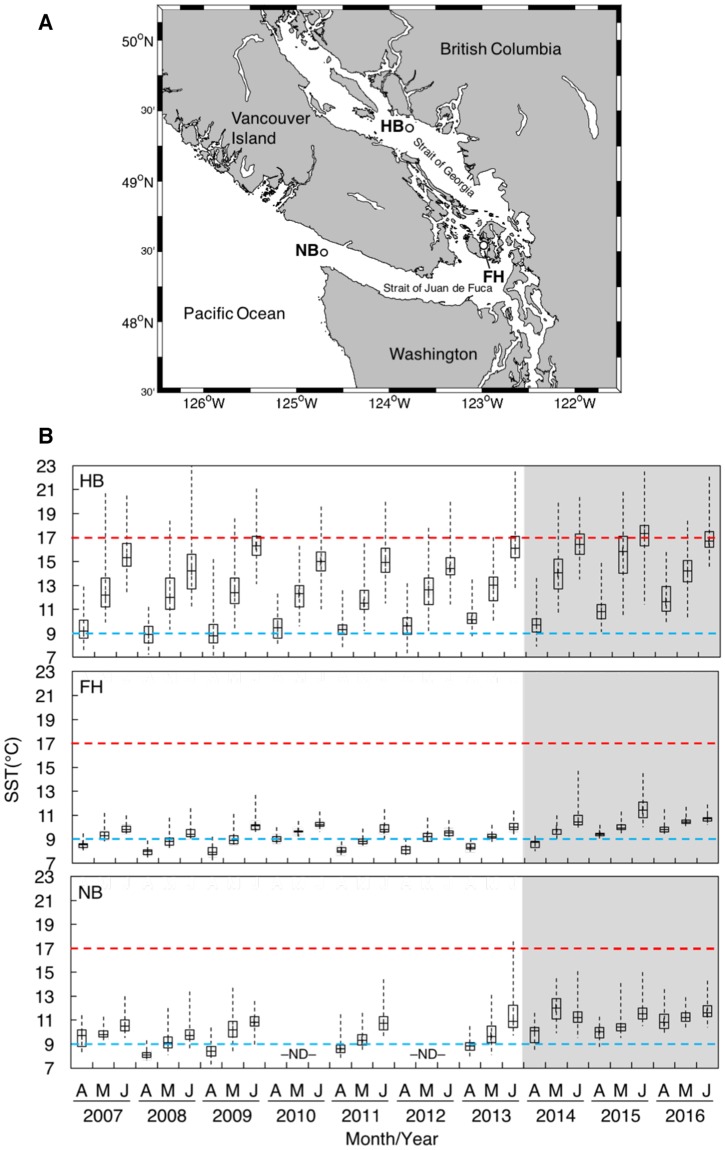
(**A**) Map of the Salish Sea on the Pacific coast of North America, showing the location of 3 oceanographic buoys (circles) where sea surface temperatures were measured (HB, Halibut Bank; FH, Friday Harbor; NB, Neah Bay). (**B**) Boxplots of sea surface temperatures (SST, °C) at the 3 buoys over 10 years (2007–2016) in the months of April–June, when larval *S. droebachiensis* are expected to be most abundant in the water column^23^. The dashed horizontal blue and red lines indicate the historical (9 °C) and warm (17 °C) temperature treatments applied to larval cultures. The grey band indicates the period of the “Blob” marine heatwave. ND = no data available.

Larvae were generated for the experiment from sea urchins collected in the Salish Sea, Washington, USA, where temperature anomalies of +4 °C were observed during the period of the warm Blob^21^. To more closely examine the effects of the Blob on sea surface temperatures (SST) in the study region, SST data were acquired from the National Oceanic and Atmospheric Administration (NOAA) National Buoy Data Center (www.ndbc.noaa.gov/) and the Department of Fisheries and Oceans Canada (DFO) Canadian Moored Buoy Historical Data (www.meds-sdmm.dfo-mpo.gc.ca/) for 3 buoy locations spanning the distribution of *S. droebachiensis* in the Salish Sea (Fig. 1A)^22^. Given that we were interested in prolonged periods of warm temperature that could affect larval development, we generated a time-series of boxplots to examine the minimum, median, and maximum temperatures in April through June of each year, when *S. droebachiensis* larvae are expected to be most abundant in the water column^23^ (Fig. 1B). We found that mean SST in April through June was significantly greater in the presence than in the absence of the Blob at all stations (one-tailed t-tests: FHL, t_2_ = 24.58, p = 0.002; HB, t_2_ = 36.34, p < 0.001; and NB, t_2_ = 20.85, p = 0.002; n = 3–7 years).

To generate larvae for the experiment, on 26 April 2016 5 adult *S. droebachiensis* (>30 mm test diameter) were injected with ~1–5 mL of 0.55 M KCl through the peristomial membrane^24^. Spawning occurred in 1 male and 2 females. A single drop of sperm diluted in 0.37 μm-filtered seawater (FSW) was used to fertilize eggs from individual females (~1 h). Resultant embryos from each parental lineage were intermixed and then transferred into 16 replicate glass culture jars with 2000 mL of FSW, at a concentration of 1 embryo mL^−1^. Larval cultures were placed in temperature-controlled chambers at either a historical (9 °C) or warm (17 °C) temperature treatment (8 cultures per temperature) and were continuously stirred at 6 RPM with motor-operated plastic paddles mounted to a plastic frame^25^. The historical treatment approximates the median sea surface temperature observed at the Friday Harbor buoy in April through June of 2007 through 2013 (Fig. 1B). The warm treatment approximates the anomalously warm median sea temperature observed at the Halibut Bank buoy in June of 2015 and 2016 (Fig. 1B). Larval cultures in each temperature treatment were fed 1 of 2 food types (phytoplankton or kelp detritus) at 1 of 2 rations (high or low), yielding 4 possible diets: phytoplankton × high ration, kelp detritus × high ration, phytoplankton × low ration, and kelp detritus × low ration. This resulted in a 3-factor fully-crossed experimental design of combined food type (2 levels), food ration (2 levels), and temperature (2 levels), with n = 2 jars per treatment combination. The phytoplankton food treatment consisted of an ~1:1 mixture (by cell volume) of *Dunaliella tertiolecta* and *Isochrysis galbana*. The phytoplankton × high ration diet consisted of 5000 cells mL^−1^ volume equivalent of *D. tertiolecta*, while the phytoplankton × low ration diet consisted of 500 cells mL^−1^ volume equivalent of *D. tertiolecta* (estimated cell volume ratio of *D. tertiolecta*:*I. galbana* of 1:8). Based on an estimated chlorophyll to biomass conversion for *D. tertiolecta* of 1 pg chlorophyll cell^−1^ ^26^, these cell concentrations represent chlorophyll concentrations of ~0.5 and 5 mg m^3^, approximating the values observed in the presence and absence of a typical phytoplankton bloom in the Salish Sea^27^. The kelp detritus food treatment consisted of particles of 1–2-week old bull kelp *Nereocystis luetkeana* suspended in FSW. To produce the detritus, fresh *N. luetkeana* blades were wiped clean and blended in an industrial blender, filtered through a 70 µm mesh to remove large particles, and aged in the dark at ambient sea temperature^20^. The kelp detritus × high ration diet consisted of 5000 kelp-derived particles mL^−1^, while the low ration diet consisted of 500 particles mL^−1^. Kelp-derived detritus can contribute up to 33% of particulate organic matter in the 20 to 63 µm size-fraction in this region^19^. A previous study has shown that suspended kelp detritus at a concentration of 5000 particles mL^−1^ rivals an optimal phytoplankton diet of *D. tertiolecta* and *I. galbana* in terms of food quality for larval *S. droebachiensis* under optimal temperature conditions^20^. Larvae were fed every 2–3 d beginning at age 5 d post-fertilization (early pluteus).

**Figure 2:**
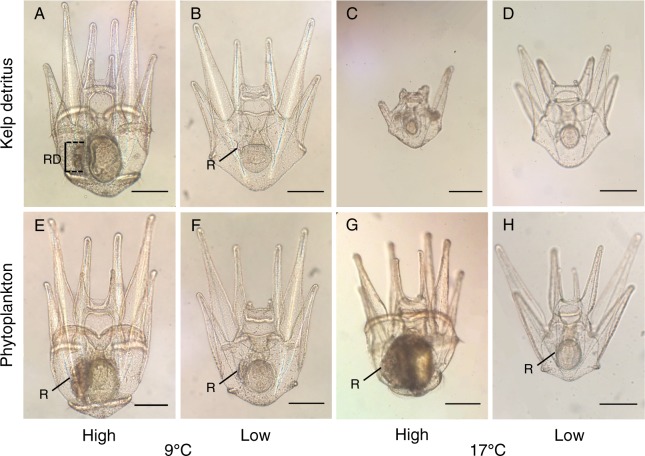
Photomicrographs of larval *Strongylocentrotus droebachiensis* at age 22 d in treatments consisting of combinations of 3 factors: food type (2 levels: kelp detritus, and phytoplankton), food ration (2 levels: high, and low), and temperature (2 levels: 9 °C, and 17 °C). In panel A: RD = echinus rudiment diameter. Rudiments (R) also are indicated in panels B and E–H. Scale bars are 200 µm.

Given that the length of the larval period often determines larval mortality rates due to exposure to predators or risk of offshore advection^28^, patterns of echinus rudiment formation in preparation for metamorphosis may be the most informative metric in predicting recruitment success of sea urchins. Rudiment diameters of larvae were measured with the ocular micrometer of a compound microscope (5 µm resolution) at ages 5, 9, 13, 16, 19 and 22 d (with d = 0 at fertilization) (Fig. 2). Two larvae were sampled without replacement from each culture jar on each sampling day. Rudiment diameter was measured at first contact of the ectodermal invagination with the hydrocoel, and acts as an indicator of juvenile sea urchin development within a larva and preparation for metamorphosis^29^.

**Figure 3:**
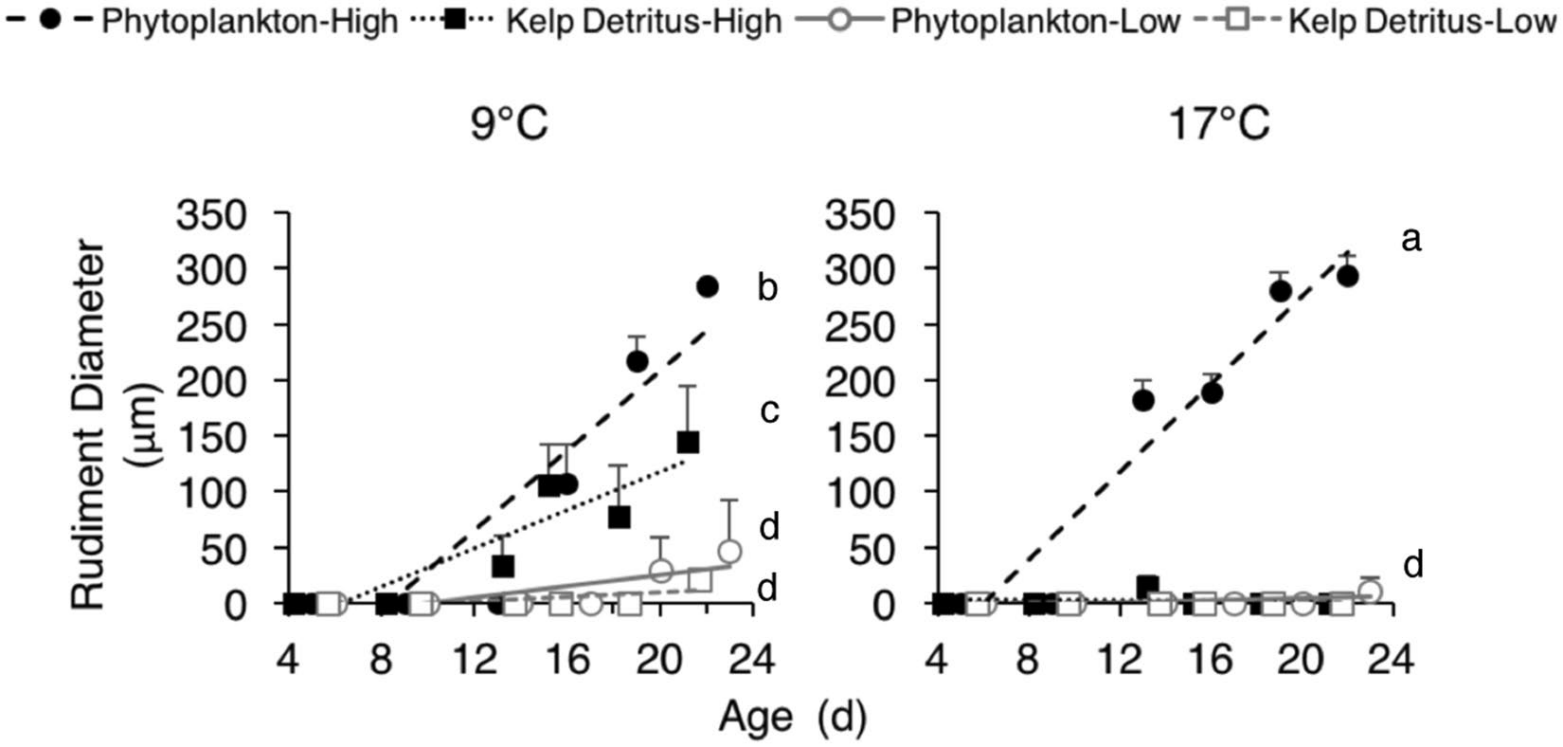
Rudiment diameters (µm) of larvae at ages 5 to 22 d (d = 0 at fertilization). Treatments are as in Fig. 2. Lines indicate linear relationships. Lowercase letters indicate statistical groupings based on paired comparisons of a significant 4-way interaction in ANCOVA (Tukey’s test, α = 0.05) (Supplementary Table S1). Error bars are +1 SE for n = 4 larvae per treatment, with some errors within the diameter of the symbols. Overlapping data points are shifted by ±1 d for visual clarity.

We detected no difference in the rate of rudiment development from age 5 to 22 d for larvae fed a low concentration of phytoplankton or a low concentration of kelp detritus at warm versus historical temperature, with limited to no rudiment formation in these treatments (Fig. 3). This indicates slow development to metamorphosis at low food concentration regardless of food type or temperature. However, at high food concentration, we observed interactive effects of food and temperature on rudiment development. Specifically, we observed faster rudiment development at warm versus historical temperature when larvae were fed a high ration of phytoplankton, and we observed the reverse pattern when larvae were fed a high ration of kelp detritus: larvae developed slower at warm versus historical temperature on a high ration kelp detritus diet (Fig. 3). ANCOVA on rudiment diameters of larvae from age 5 to 22 d showed a significant 4-way interaction between food type, food ration, temperature, and age (covariate), indicating heterogeneity of slopes and an interactive effect of treatments on rudiment development rates (Supplementary Table S1). Paired comparisons of treatments with Tukey’s test (α = 0.05) indicated that the slope was significantly greater for the high ration phytoplankton treatment at 17 °C versus 9 °C (Fig. 3, lowercase letters indicate statistical groupings). Both of these slopes were significantly greater than the slopes of all other treatments. At 17 °C, there was no difference in slopes between the high ration kelp detritus, low ration kelp detritus, and low ration phytoplankton treatments. At 9 °C, the slope of the high ration kelp detritus treatment was significantly greater than the slopes of the low ration kelp detritus and low ration phytoplankton treatments, which did not differ from one another. Additionally, the slope of the high ration kelp detritus treatment was significantly greater at 9 than 17 °C. The slope of the high ration kelp detritus treatment at 9 °C also was significantly greater than the slope of the low ration phytoplankton and low ration kelp detritus treatments at 17 °C. Slopes did not differ among low ration food treatments at 9 versus 17 °C (Fig. 3). These results indicate that a high concentration of phytoplankton mitigated a negative effect of warm temperature on larval development (antagonistic effect), while a high concentration of kelp detritus exacerbated the negative effect of warm temperature on larval development (synergistic effect). The former result suggests that in the presence of abundant phytoplankton, larvae have sufficient energy to compensate for increased metabolic requirements at warm temperature. This interpretation is consistent with a broadly accepted positive relationship between temperature and metabolic energy requirements of marine invertebrates^30^. In an analogous finding, Yeakel *et al*.^31^ observed that tropical corals can achieve high rates of calcification at suboptimal (low) pH when they have access to a nutritionally replete diet. Given that conditions of warm temperature and high phytoplankton concentration did not to our knowledge co-occur in the field during the 2013–2016 marine heatwave (to the contrary, phytoplankton were likely rare; 4), our results should be interpreted with caution. It is likely that a combination of anomalously warm sea temperature and low phytoplankton concentration during the heatwave led to slow development of sea urchin larvae, delaying metamorphosis.

In addition to slow development of larvae in the warm, high ration kelp detritus treatment, there was complete mortality of larvae in this treatment by day 26 (Supplementary Table S2). Following the observation of complete mortality in the warm, high ration kelp detritus treatment, we measured dissolved oxygen in all larval cultures on day 26 (YSI Model PRO 2030) and found lower dissolved oxygen in this treatment (DO = 96 ± 1%, mean ± SD, n = 2) as compared to all other treatments (DO = 101 ± 2%, n = 14). While this does not provide evidence that the warm, high ration kelp detritus cultures were hypoxic, it does suggest higher rates of respiration in this treatment due to microbes. We posit that opportunistic deleterious microbes associated with the kelp detritus bloomed in this treatment. The presence of deleterious microbes could have contributed to slow growth of larvae due to the energetic cost of mounting an immune response. This hypothesis assumes that deleterious microbes were most abundant in the warm, high ration kelp detritus treatment due to a higher dosage of microbes (i.e. greater volume of kelp detritus added as compared to the low ration diet) and faster growth of microbes at warm versus historical temperature. It is also possible that microbial films on the insides of the jars differed among the kelp detritus and phytoplankton treatments. However, these inferences remain equivocal, as we did not investigate the microbial composition of the kelp detritus before or after addition to the larval cultures. In natural ocean environments, regions of high nutrient concentration have a high concentration of associated deleterious microbes that pose a risk to developing marine invertebrate larvae^32^. In accordance, in laboratory cultures, the microbiome of larval *S. droebachiensis* under food-rich conditions shifts towards a state of dysbiosis, leading to mortality^32^. An alternate hypothesis for our observations is that large microbes inedible to larvae, such as ciliates, bloomed in the warm, high ration kelp detritus cultures, reducing larval feeding efficiency and causing starvation. Decreased feeding efficiency can result from low clearance rates of larvae due to time spent rejecting large, inedible particles^33^. Previous studies have shown that the presence of large inedible particles reduces larval feeding efficiency in a dosage-dependent manner^33,34^.

**Figure 4:**
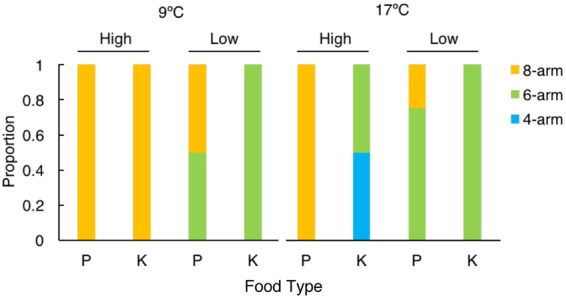
Proportion of larvae at the 4-, 6-, and 8-arm stage at age 22 d. Treatments are as in Fig. 2. P = Phytoplankton, and K = Kelp detritus. Proportions are based on 4 larvae per treatment.

At age 22 d, larval arms were counted for classification of larval stage (4-arm, 6-arm, and 8-arm pluteus) in each treatment. Larval developmental stages observed at age 22 d yielded patterns similar to the results for rudiment development. The high ration kelp detritus and phytoplankton diets resulted in comparable development at historical temperature, with all larvae reaching the 8-arm stage (Fig. 4). However, at warm temperature, larvae in the high ration phytoplankton treatment all reached the 8-arm stage, while larvae in the high ration kelp detritus treatment were less developed, at the 4-arm or 6-arm stage (Fig. 4). This result is consistent with a synergistic negative effect on larval development of combined warm temperature and a high ration kelp detritus diet.

Suspended kelp-derived detritus has been documented in high abundance along coasts in the Northeast Pacific^18,19^ and may be an alternate food source (to phytoplankton) for larval sea urchins^20^. However, little is known about the microbial communities that colonize and metabolize this material during the kelp degradation process^20^. We found that a high concentration of kelp detritus did not mitigate thermal stress in sea urchin larvae in the laboratory, but rather, synergistically reduced larval development and survival when combined with warm temperature. Additional research is needed to determine whether (and how) microbial communities associated with kelp detritus contribute to slow growth and high mortality of larvae, and whether our laboratory observations are representative of interactions between larvae and their food sources in the field.

## Electronic supplementary material

Supplementary Information File

## Data Availability

Figure 1 has associated raw data publicly available from www.ndbc.noaa.gov and www.meds-sdmm.dfo-mpo.gc.ca. All other data analyzed are included in this article (and its supplementary information files).
