# Supplementary material for: Synergistic negative effects of thermal stress and altered food resources on echinoid larvae
Source: Sci Rep. 2018 Aug 15;8:12229. doi: 10.1038/s41598-018-30572-w (PMC6093897; doi:10.1038/s41598-018-30572-w)
Supplement: Supplementary file 1 — Supplementary Information File [file 41598_2018_30572_MOESM1_ESM.pdf]

Supplementary Information for

Synergistic negative effects of thermal stress and altered food resources on echinoid larvae

Colette J. Feehan, Zoe Ludwig, Suzannah Yu, and Diane K. Adams

PDF contains:

Table S1, and S2

Methods

References

Table S1. Analysis of covariance (ANCOVA) of the effect of food type (‘Type’, fixed factor, 2 levels: phytoplankton, kelp detritus), food ration (‘Ration’, fixed factor, 2 levels: high, low), temperature (‘Temp’, fixed factor, 2 levels: 9 and 17ºC), larval age (‘Age’, covariate), and jar nested within Type × Ration × Temp on rudiment diameters (µm) of larval *Strongylocentrotus droebachiensis*. Based on a highly non-significant effect of the nested factor, jars were pooled within treatments to increase the degrees of freedom of the analysis (n = 4 larvae) [35]. Significant values at α = 0.05 are shown in bold.

Effect	df	MS	F	p
Type	1	2.4×10^4	16.6	**<0.001**
Ration	1	3.8×10^4	26.5	**<0.001**
Temp	1	5.8×10^3	4.1	**0.046**
Age	1	2.5×10^5	171.2	**<0.001**
Type × Ration	1	1.7×10^4	11.6	**<0.001**
Type × Temp	1	3.7×10^2	0.3	0.612
Ration × Temp	1	1.7×10^3	1.2	0.284
Type × Age	1	9.6×10^4	66.9	**<0.001**
Ration × Age	1	1.8×10^5	123.3	**<0.001**
Temp × Age	1	8.7×10^3	6.1	**0.015**
Type × Ration × Temp	1	1.2×10^3	0.8	0.373
Type × Ration × Age	1	7.2×10^4	49.9	**<0.001**
Type × Temp × Age	1	9.1×10^3	6.3	**0.013**
Ration × Temp × Age	1	1.2×10^3	0.9	0.356
Type × Ration × Temp × Age	1	1.5×10^4	10.1	**0.002**
Jar (Type × Ration × Temp)	8	1.0×10^3	0.7	0.685
Residual	176	1.4×10^3		

Table S2. Survival (%; mean ± SD) of larval *Strongylocentrotus droebachiensis* at age 26 days in treatments in Fig. 2 (n = 2 jars). ND = No data available due to settlement of larvae.

Treatment			Survival (%; mean ± SD)
**Temperature**	**Food Ration**	**Food Type**	
9°C	High	Phytoplankton	ND
	High	Kelp detritus	36 ± 24
	Low	Phytoplankton	54 ± 14
	Low	Kelp detritus	46 ± 9
17°C	High	Phytoplankton	ND
	High	Kelp detritus	0 ± 0
	Low	Phytoplankton	39 ± 11
	Low	Kelp detritus	56 ± 9

Methods

Sea surface temperatures

Changing thermal conditions in the Salish Sea could alter sea urchin recruitment success through effects on larval growth, development, and survival. To examine the effect of the warm Blob on sea surface temperatures (SST) in the Salish Sea, SST data with 6 min sampling frequency were acquired from the National Oceanic and Atmospheric Administration (NOAA) National Buoy Data Center (www.ndbc.noaa.gov/) for the Friday Harbor buoy (FH; Station FRDW1), ~2.5 km WSW of the site of collection of *Strongylocentrotus droebachiensis* spawned for the larval experiment (see Larval culture), and the Neah Bay buoy (NB; Station 46087), ~120 km W of the collection site (Fig. 1). Additional SST data with hourly sampling frequency were acquired from the Department of Fisheries and Oceans Canada (DFO) Canadian Moored Buoy Historical Data (www.meds-sdmm.dfo-mpo.gc.ca/) for the Halibut Bank buoy (HB; Station 14146), ~120 km NW of the collection site (Fig. 1). These buoy stations span the distribution of *S. droebachiensis* in the Salish Sea [22]. Data over 10 years (2007–2016) were analyzed for April through June, when *S. droebachiensis* larvae are expected to be most abundant in the water column [23]. Average SST in April through June was significantly greater in the presence (2014–2016) than in the absence (2007–2013) of the warm Blob at all stations (one-tailed t-tests: FHL, $t_2 = 24.58$, $p = 0.002$; HB, $t_2 = 36.34$, $p < 0.001$; and NB, $t_2 = 20.85$, $p = 0.002$; $n = 3–7$ years). Note that the Blob was first observed in the winter of 2013/14. Given that we were interested in prolonged periods of warm temperature that could affect larvae, we generated a time-series of boxplots to examine the minimum, median, and maximum temperatures in individual months (April–June) within each year.

Larval culture

Reproductively mature *S. droebachiensis* used in the study were collected by divers with SCUBA from a *Nereocystis luetkeana* kelp bed on the southwestern shore of Shaw Island, WA (48.56°N, 122.99°W) on 13 April 2016. Prior to spawning, sea urchins were maintained in a flow-through seawater system at Friday Harbor Laboratories, San Juan Island, WA and fed kelp *N. luetkeana*. On 26 April 2016 5 *S. droebachiensis* were injected with ~1–5 mL of 0.55 M KCl through the peristomial membrane [24]. Spawning occurred in 1 male and 2 females. A single drop of sperm diluted in 0.37 µm-filtered seawater (FSW) was used to fertilize eggs from individual females (~1 h). Resultant embryos from each parental lineage were intermixed and then transferred into 16 replicate glass culture jars with 2000 mL of FSW, at a concentration of 1 embryo mL^{-1}. Larval cultures were continuously stirred at 6 RPM with motor-operated plastic paddles mounted to a plastic frame [25].

Temperature and food treatments

Larval cultures were placed in temperature-controlled chambers at either a historical (9°C) or warm (17°C) temperature treatment (8 cultures per temperature). The historical treatment approximates the median sea surface temperature observed at the Friday Harbor buoy in April through June of 2007 through 2013 (Fig. 1). The warm treatment approximates the anomalously warm median sea temperature observed at the Halibut Bank buoy in June of 2015 and 2016 (Fig. 1). The environmental chambers had a fluorescent light regime of 12 h light : 12 h dark. Mean (± SD) temperature in the environmental chambers was monitored during the experiment with TidbiT v2 Data Loggers (Onset Computer Corporation): 8.9 ± 0.6°C and 16.9 ± 0.7°C (n = 26 days) in the historical and warm treatment, respectively.

Larval cultures in each temperature treatment were fed 1 of 2 food types (phytoplankton or kelp detritus) at 1 of 2 rations (high or low), yielding 4 possible diets: phytoplankton × high ration, kelp detritus × high ration, phytoplankton × low ration, and kelp detritus × low ration. This resulted in a 3-factor fully-crossed experimental design of combined food type (2 levels), food ration (2 levels), and temperature (2 levels), with n = 2 jars per treatment combination. The phytoplankton food treatment consisted of an ~1:1 mixture (by cell volume) of *Dunaliella tertiolecta* and *Isochrysis galbana*. The phytoplankton × high ration diet consisted of 5000 cells mL^{-1} volume equivalent of *D. tertiolecta*, while the phytoplankton × low ration diet consisted of 500 cells mL^{-1} volume equivalent of *D. tertiolecta* (cell volume ratio of *D. tertiolecta* : *I. galbana* of 1 : 8, i.e. 8 times the number of *I. galbana* as *D. tertiolecta* cells were added to achieve a 1:1 volume ratio for these two species). Based on an estimated chlorophyll to biomass conversion for *D. tertiolecta* of 1 pg chlorophyll $cell^{-1}$ [26], these cell concentrations represent chlorophyll concentrations of ~ 0.5 and 5 mg m^3, approximating the values observed in the presence and absence of a typical phytoplankton bloom in the Salish Sea [27]. Phytoplankton were grown at room temperature in Guillard's f/2 nutrient medium with constant fluorescent illumination. The kelp detritus food treatment consisted of particles of 1–2-week old *N. luetkeana* detritus suspended in FSW. To produce the detritus, fresh *N. luetkeana* blades were wiped clean and blended in an industrial blender, filtered through a 70 µm mesh to remove large particles, and aged in the dark at ambient sea temperature [20]. The kelp detritus × high ration diet consisted of 5000 kelp-derived particles mL^{-1}, while the low ration diet consisted of 500 particles mL^{-1}. Kelp-derived detritus can contribute up to 33% of particulate organic matter in the 20 to 63 µm size-fraction in this region [19]. A previous study has shown that suspended kelp detritus at a concentration of 5000 particles mL^{-1} rivals a high ration phytoplankton diet in terms of food

quality for *S. droebachiensis* larvae under optimal temperature conditions [20]. Phytoplankton and kelp detritus concentrations were determined with a Guava easyCyte flow cytometer (Millipore EMD). Larvae were fed every 2 to 3 days beginning at age 5 days post-fertilization (early pluteus stage). Prior to each feeding, 75% of the water in each culture jar was replaced with fresh FSW. Culture jars were cleaned prior to feeding by rinsing with ~1000 mL of FSW.

Morphological measurements

Rudiment diameters of larvae were measured with the ocular micrometer of a compound microscope (5 μm resolution) to monitor development in treatments at ages 5, 9, 13, 16, 19 and 22 days (with d = 0 at fertilization) (Fig. 2). Two larvae were sampled without replacement from each culture jar on each sampling day. At 22 days, larval arms were counted for classification of larval stage (4-arm, 6-arm, and 8-arm pluteus). Rudiment diameter was measured at first contact of the ectodermal invagination with the hydrocoel, and acts as an indicator of juvenile sea urchin development within a larva and preparation for metamorphosis [29]. Sampling of larvae without replacement reduced concentrations in the cultures by <1 %.

The experiment was terminated after 26 days, following observation of settlement of ~50% of larvae into benthic juveniles in both replicates of the 9 and 17°C phytoplankton × high ration treatments. At this time, larval concentrations were measured in remaining cultures in which settlement had not yet occurred by removing 25 mL of culture water and enumerating larvae under a dissecting microscope (n = 2). Percent survival of larvae was calculated as larval concentration at termination of the experiment divided by larval concentration at the onset of the experiment (corrected for larvae removed for body measurements) multiplied by 100%.

Statistical Analysis

To examine the combined effects of temperature and food treatments on development of larvae, we used analysis of covariance (ANCOVA) to test for homogeneity of slopes of rudiment diameter vs. age for treatments from age 5 to 22 days. The analysis was conducted on rudiment diameter (μm), with age as the covariate, and food ration (2 levels, fixed factor: high and low), food type (2 levels, fixed factor: kelp detritus and phytoplankton), and temperature (2 levels, fixed factor: 9 and 17°C) as categorical predictors. To test for jar effects in ANCOVA, we included an additional factor of jar nested within the interaction among food type, food ration, and temperature. Where the nested factor was highly non-significant, jars were pooled within treatments to increase the degrees of freedom of the analysis [35]. The assumption of homogeneity of variance for ANCOVA was confirmed with Cochrane's C-test ($\alpha = 0.05$). Post hoc analysis of factors significant in ANCOVA was conducted with Tukey's HSD test ($\alpha = 0.05$). Data were analyzed with Statistica 13.2 (StatSoft).

References

35. Strathmann, R.R., Fenaux, L., Sewell, A.T. & Strathmann, M.F. Abundance of food affects relative size of larval and postlarval structures of a molluscan veliger. *Biol. Bull.* **185**, 232–239 (1993).
